# Overview of Developments in the MRCC Program System

**DOI:** 10.1021/acs.jpca.4c07807

**Published:** 2025-02-17

**Authors:** Dávid Mester, Péter R. Nagy, József Csóka, László Gyevi-Nagy, P. Bernát Szabó, Réka A. Horváth, Klára Petrov, Bence Hégely, Bence Ladóczki, Gyula Samu, Balázs D. Lőrincz, Mihály Kállay

**Affiliations:** † Department of Physical Chemistry and Materials Science, Faculty of Chemical Technology and Biotechnology, Budapest University of Technology and Economics, Műegyetem rkp. 3., H-1111 Budapest, Hungary; ‡ HUN-REN-BME Quantum Chemistry Research Group, Műegyetem rkp. 3., H-1111 Budapest, Hungary; § MTA-BME Lendület Quantum Chemistry Research Group, Műegyetem rkp. 3., H-1111 Budapest, Hungary

## Abstract

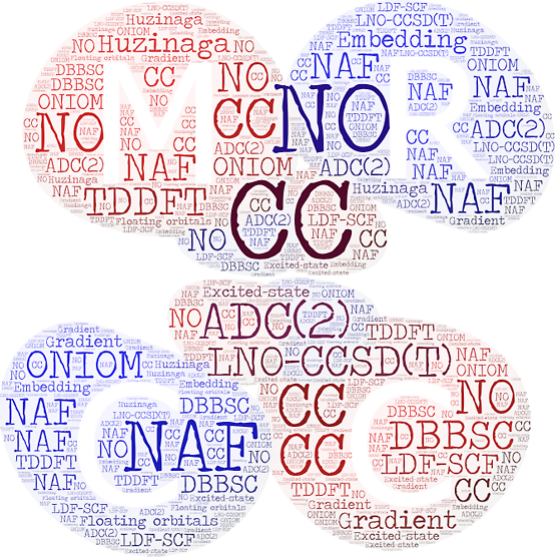

mrcc is a versatile suite of quantum chemistry programs designed for accurate *ab initio* and density functional theory (DFT) calculations. This contribution outlines the general features and recent developments of the package. The most popular features include the open-ended coupled-cluster (CC) code, state-of-the-art CC singles and doubles with perturbative triples [CCSD(T)], second-order algebraic-diagrammatic construction, and combined wave function theory-DFT approaches. Cost-reduction techniques are implemented, such as natural orbital (NO), local NO (LNO), and natural auxiliary function approximations, which significantly decrease the computational demands of these methods. This paper also details the method developments made over the past five years, including efficient schemes to approach the complete basis set limit for CCSD(T) and the extension of our LNO-CCSD(T) method to open-shell systems. Additionally, we discuss the new approximations introduced to accelerate the self-consistent field procedure and the cost-reduction techniques elaborated for analytic gradient calculations at various levels. Furthermore, embedding techniques and novel range-separated double-hybrid functionals are presented for excited-state calculations, while the extension of the theories established to describe core excitations and ionized states is also discussed. For academic purposes, the program and its source code are available free of charge, and its commercial use is also facilitated.

## Introduction

mrcc is a suite of *ab initio* and density functional quantum chemistry programs designed for efficient and high-precision electronic structure calculations.^1^ The development of this program package, which now offers a wide range of functionalities, began in 2000 at the Eötvös University, Hungary. The primary goal of the research conducted at that time was to elaborate an automated string-based technique facilitating the implementation of arbitrary-order coupled-cluster (CC) methods.^2^ The development of the code was continued at the University of Mainz, Germany, between 2002 and 2004. The analytic gradients developed during this period enabled accurate calculations of physical and chemical properties at the highest levels of wave function theory (WFT), specifically within the framework of CC and configuration interaction (CI) theories.^3−8^ From 2004, the program’s functionality has been expanded at the Budapest University of Technology and Economics, resulting in mrcc becoming a versatile standalone software. The code has been officially distributed since 2005, and the number of registered users has grown dynamically, exceeding 1100 today. Over the past 15 years, the relatively small group, comprising 5 to 10 members, has primarily consisted of PhD students and early career postdoctoral researchers, who have been the primary contributors to the software and method development. The software development has been carried out in Fortran.

The development of the program has always been driven by the goal of efficiently performing high-precision calculations. The first step in this direction was the development of an automated string-based algorithm that enabled arbitrary-order CC and CI calculations. This infrastructure was later extended to include perturbative approximations,^9,10^ multireference methods,^11,12^ and the calculation of excited states^13^ and analytic gradients.^3^ Following this, the focus shifted to accurate calculations for larger molecules, resulting in highly optimized implementations for lower-order methods. Our research required the development of an efficient electron repulsion integral (ERI) code^14,15^ and a self-consistent field (SCF) program^16^ utilizing the density fitting (DF) approximation. Simultaneously, efficient and state-of-the-art codes for correlation methods such as MP2,^17^ dRPA,^18^ CCSD(T),^16,19,20^ CIS(D),^21^ and ADC(2)^22^ (see Tables 1 and 2 for the meaning of acronyms) were developed, and the software was also adapted for density functional theory (DFT) calculations.

**Table 1:** Methods Implemented in mrcc for Ground-State Calculations^d^

	method	references		
abbreviation	full name/description	method^a^	implementation^b^	gradient^c^
HF	Hartree–Fock	(38 and 39)	(16)	+
MCSCF	multiconfigurational self-consistent field	(40)		–
KS	Kohn–Sham	(41 and 42)	(18)	+
DH-DFT	double-hybrid density functional theory	(43)	(31 and 32)	+
MP2	second-order Møller–Plesset (MP) perturbation theory	(44–46)	(17)	+
MP2-F12	explicitly correlated MP2	(47 and 48)	(49)	–
MP3	third-order MP perturbation theory	(50)	(20)	–
dRPA	direct random-phase approximation	(51)	(17 and 18)	–
SOSEX	second-order screened exchange	(52)	(18)	–
rPT2	renormalized second-order perturbation theory	(53)	(54)	–
RPA	random-phase approximation	(55)	(18)	–
RPAX2	approximate RPA with exchange	(56)	(31)	–
CC2	approximate CC singles and doubles	(57–59)	(29)	–
CCSD	CC singles and doubles	(60 and 61)	(16, 19, and 20)	–
CCSD(T)	CCSD with perturbative triples	(62)	(16, 19, and 20)	–
CCSD(F12*)	explicitly correlated CCSD using the CCSD(F12*) ansatz	(63)	(49)	–
CCSD(F12*)(T+)	CCSD(F12*) with the (T+) correction	(49 and 63)	(49)	–
				
CC(*n*)	CC method including up to *n*-tuple excitations	(2)	(2)	+
CC(*n* – 1)(*n*)	CC(*n* – 1) method with perturbative *n*-tuple excitations	(9 and 10)	(9)	–
CC(*n* – 1)[*n*]	generalization of CCSD[T]^64^ to *n*-tuple excitations	(9 and 10)	(9 and 10)	–
CC(*n* – 1)(*n*)_Λ_	generalization of CCSD(T)_Λ_^65,66^ to *n*-tuple excitations	(9)	(9)	–
CC*n*	generalization of CC3^67^ to *n*-tuple excitations	(9)	(9)	–
CI(*n*)	CI method including up to *n*-tuple excitations	(38)	(2)	+
FCI	full CI	(38)	(2)	+
MRCC(*n*)	multireference CC(*n*)	(11 and 68)	(11)	+
MRCI(*n*)	multireference CI(*n*)	(11 and 69)	(11)	+

a References describing the methodological developments.

b References describing the implementation in mrcc.

c Availability of analytic gradients.

d The upper panel represents the hand-coded, highly-optimized implementations, while the lower panel shows those using automated string-based techniques.

**Table 2:** Methods Implemented in mrcc for Excited-State Calculations^d^

	method	references		
abbreviation	full name/description	method^a^	implementation^b^	gradient^c^
CIS	configuration interaction singles	(77)	(28)	+
MCSCF	multiconfigurational self-consistent field	(40)		–
TDHF	time-dependent Hartree–Fock	(78)	(28)	–
(TDA-)TDDFT	(Tamm–Dancoff approximation) TDDFT	(79 and 80)	(28)	–
DH-TDDFT	double-hybrid TDDFT	(36, 81)	(36)	–
CIS(D)	CIS with perturbative doubles	(82–84)	(21)	–
CIS(D_∞_)	CIS with iterative approximate doubles	(58, 59, and 85)	(29)	–
ADC(2)	second-order algebraic-diagrammatic construction	(58, 59, and 86)	(22)	–
LR-CC2	linear response CC2	(57–59)	(29)	–
				
LR-CC(*n*)	linear response CC(*n*)	(2)	(2 and 13)	+
CI(*n*)	CI method including up to *n*-tuple excitations	(38)	(2)	+
FCI	full CI	(38)	(2)	+
LR-MRCC(*n*)	linear response MRCC(*n*)	(11 and 68)	(11 and 13)	+
MRCI(*n*)	multireference CI including up to *n*-tuple excitations	(11 and 69)	(11)	+

a References describing the methodological developments.

b References describing the implementation in mrcc.

c Availability of analytic gradients.

d The upper panel represents the hand-coded, highly-optimized implementations, while the lower panel shows those using automated string-based techniques.

Remaining true to the original ideology, our research over the past decade has focused on reducing computational requirements for accurate electron correlation approaches and further improving the accuracy of DFT methods. Accordingly, efficient cost-reduction techniques have been developed for correlation methods,^16−19,22−30^ and accurate combined WFT-DFT approaches have been proposed.^31−36^ During the development, we always kept modularity, user-friendliness, and the cost-to-performance ratios in mind. Consequently, the high flexibility of the implementations allows for the rapid development and testing of new methods, while the highly optimized algorithms provide remarkable performance. Our current and future goals are to push the limits further regarding the range of physical and chemical properties that can be computed and the size of systems that can be studied. To achieve this, we aim to combine cost-reduction techniques with the calculation of analytic gradients, enabling the determination of accurate properties for large molecules within a reasonable computational time frame, as well as to further enhance the robustness and accuracy of combined WFT-DFT methods.

This contribution presents the features of the mrcc program package. In a similar publication in 2020,^37^ we discussed the methods available at that time and their performance in detail. Therefore, in this paper, we primarily focus on the developments made over the past five years. First, the general features are briefly discussed, highlighting the most popular ones. Subsequently, a detailed discussion of the new advancements is provided. In particular, our novel schemes to approach the complete basis set (CBS) limit for the CCSD(T) method are considered, and the extension of our linear-scaling correlation methods to open-shell systems is presented. Additionally, our efficient approximations and algorithms for SCF, excited-state methods, and analytic gradients are also discussed.

## Results and Discussion

### General Features

#### Ground-State Calculations

Here, we briefly discuss the general and most popular features of mrcc, organized by different types of methods. The list of currently available ground-state methods is presented in Table 1.

To generate HF molecular orbitals (MOs), mrcc provides a highly efficient SCF program using both disk-based and integral-direct implementations. The optimized infrastructure is equipped with standard convergence acceleration techniques, such as direct inversion in the iterative subspace (DIIS), damping techniques, and level shifting. The procedure can start from various initial guesses or restarted from smaller basis set calculations. Quadratic SCF algorithms were also written based on Newton and quasi-Newton schemes, while an MCSCF procedure was also developed supporting complete active spaces.

The same infrastructure can be used for KS calculations, where local density approximation, generalized gradient approximation (GGA), meta-GGA, hybrid, range-separated (RS) hybrid, and van der Waals functionals are available. To extend the applicability, an interface was developed to the libxc library of density functionals,^70−72^ which enables the use of several hundred functionals, while empirical dispersion corrections can also be calculated using the DFT-D3 scheme.^73,74^ Additionally, mrcc offers a wide range of post-KS approaches. Specifically, the most common MP2-based DHs are available,^43^ while nonconventional post-KS methods were also implemented based on dRPA and its improved variants, such as SOSEX, RPAX2, and rPT2. Enhancing efficiency, the DF approximation is extensively utilized in these implementations, and spin-scaled and RS variants can also be defined.^21,31−33,75^ The flexible and user-friendly framework facilitates the development of new post-KS approaches.

The program offers a variety of *ab initio* electron correlation methods. The original focus of the program development was on higher-order CC and CI methods using automated string-based techniques. Accordingly, several theories are available up to arbitrary excitations, such as the standard CC(*n*) and CI(*n*) approaches, as well as the CC(*n* – 1), CC[*n* – 1] and CC(*n* – 1)(*n*)_Λ_ methods, which include perturbative approximations. Utilizing this infrastructure,^2,9,10^ general state-selective MRCC and MRCI approaches have also been elaborated. In addition to energy calculations, other properties can be calculated analytically for general CC methods, such as dipole and higher moments,^3^ vibrational frequencies, NMR chemical shifts,^76^ magnetizabilities,^4^ static and frequency-dependent polarizabilities,^5^ and Raman intensities.^8^ Furthermore, in recent years, a highly optimized standard and explicitly correlated CCSD(T) implementation has been developed, relying on the DF approximation.^20,49^

For most of the methods, any kind of MOs—restricted, unrestricted, and restricted open-shell HF (ROHF) and KS (ROKS) orbitals—can be used. Additionally, analytic gradients can be calculated for single- and multireference CC(*n*) and CI(*n*) methods, while they are also available for the HF, KS, and MP2 approaches.

#### Excited-State Calculations

Excited-state calculations can also be performed with mrcc. The available methods are listed in Table 2.

The automated programming tools have been extended to include excited-state theory, making these calculations available for all nonperturbative approaches, such as single- and multireference LR-CC(*n*) and CI(*n*) methods. Additionally, utilizing the DF approximation, very fast first- and second-order excited-state methods including CIS, (TDA-)TDDFT, CIS(D), LR-CC2, and ADC(2) have been implemented. In these cases, both disk-based and integral-direct algorithms were developed. Flexible spin-scaling techniques are also available for the spin-component-scaled (SCS) second-order methods,^58,83,84^ and an *N*^4^-scaling algorithm has been written for the iterative scaled-opposite-spin (SOS) approaches utilizing a Laplace transform-based procedure.^59^ For TDDFT calculations, pure, hybrid, and DH functionals are available. Furthermore, for DH calculations, both CIS(D)- and ADC(2)-based DH ansätze can be selected.^36,81^ To visualize the state-specific orbitals, natural transition orbitals can be calculated,^87^ while Dyson orbitals are also available for ADC(2)-based electron-attached/detached states.^88^

Ground to excited-state transition moments can be evaluated with all the methods listed in Table 2, except for MCSCF and LR-CC2, while spectral intensities, including oscillator and rotator strengths in both the length and velocity gauges, are also available. For the string-based implementations, any kind of orbitals can be used, while the first- and second-order methods are available only for closed-shell systems. Analytic gradients are implemented for the CIS and single- and multireference LR-CC(*n*) and CI(*n*) methods. Optimization of molecular geometries is possible with any ground- and excited-state method implemented in mrcc for which analytic gradients are available (see Tables 1 and 2).

#### Reduced-Cost and Reduced-Scaling Techniques

Over the past decade, a significant focus of our research has been on accelerating electronic structure calculations. To this end, we have developed and adapted several techniques that effectively speed up various computational methods. The theory and performance of these approaches is well-documented in the literature (see Table 4 for references); therefore, only a brief theoretical background for each method will be presented here.

First, approximations for ground-state calculations are discussed. An efficient method for accelerating SCF calculations is the local DF (LDF) approximation,^26,89^ which utilizes that spatially localized electron density distributions can be expanded in local auxiliary function bases. In this approach, the MOs are localized in each SCF step, and a local fitting domain is constructed, containing only the atoms and their associated auxiliary functions necessary for the accurate fitting of the Coulomb integrals of the given localized MO (LMO). This approximation formally reduces the quartic scaling of the exchange computation to cubic; however, the scaling can be reduced to even linear if further approximations are employed,^90,91^ which were also implemented in mrcc. Additionally, for HF and KS calculations, the dual basis set approach is recommended,^35,92^ where the SCF energy, density matrix, and MOs obtained with a smaller basis set are projected into a larger one. Subsequently, the approximate large-basis SCF energy is obtained in a single step, thereby reducing the time required for the iterations.

For implementations relying on the DF approximation, the time and memory requirements of the calculations can be effectively reduced by applying the natural auxiliary function (NAF) approach,^17^ where a rank-reduced representation of the ERI tensor is used. In this case, the size of the auxiliary basis set can be effectively reduced via the singular value decomposition (SVD) of the three-center integral matrix. Even greater speedups can be achieved with orbital transformation techniques, where the MOs undergo an appropriate transformation and the resulting basis is truncated. One of the most effective approaches is the natural orbital (NO) approximation,^93,94^ where a one-particle density matrix is constructed using a more approximate method, such as MP2. Thereafter, the matrix is diagonalized, the resulting NOs with small occupation numbers are neglected, and the expensive equations are solved only in the reduced basis. It is worth noting that the NAF and NO approximations can be freely combined effectively, as even fewer NAFs are required with the compressed NO basis.

Local approximations can also effectively reduce the computational expenses of electron correlation methods by exploiting the rapid decay of electron–electron interactions with distance. By transforming delocalized canonical orbitals obtained from SCF calculations to LMOs, the disparity in the significance of electron interactions across the molecule can be exploited.^95^ In our approach, the correlation energy is decomposed into the sum of contributions from individual LMOs,^96^ a compact domain is constructed around each LMO that includes all the significant interactions, and the corresponding contributions are evaluated only within these restricted domains.^23^ The missing interactions are accounted for by second-order pair correlation energies utilizing multipole approximations.^24^ Thereby, the number of wave function parameters and integrals can be drastically reduced, while the error in the final result remains negligible.^25,97^ Furthermore, for post-MP2 methods, the calculation of contributions within the domain can be further accelerated by using local NOs (LNOs) and NAFs.^24^ The domain-based framework is currently available for the MP2, dRPA, DH-DFT, and CC(*n*) and CC(*n* – 1)(*n*) methods using closed-shell and ROHF or ROKS references. Among these, the local MP2 (LMP2) and, in particular, the LNO-CCSD(T) approach have attracted exceptional interest.^97^ Besides its remarkable accuracy, the LNO-CCSD(T) implementation achieves asymptotically linear-scaling operation counts, constant memory and disk space requirements, and minimizes input/output (I/O) operations, making it applicable even to large proteins with thousands of atoms.^24,25^ The independence of domain constructions allows for efficient parallel execution, with potential speedups through point group symmetry identification. The black-box approach is completely *ab initio*, with no reliance on empirical methods or user decisions, and is optimized for both single workstations and large computer clusters. The key computational aspects and features of the LNO-based methods are summarized in Table 3 and explained in the section below introducing our open-shell local correlation approaches. A more detailed summary of the accuracy and efficiency of LNO-CCSD(T), along with a review of its 50+ recent applications across main group, transition metal, surface, and biochemistry, can be found in ref (97).

**Table 3:** Summary of the Advanced Features of the LNO-Based Methods (Left) and the Corresponding Computational Benefits (Right)

approaches of LNO methods	resulting benefit
approximations adapt to the wave function, i.e., no fragmentation, bond breaking, real space cutoff	systematically converging LNO settings series and extrapolation toward canonical limit
LNOs, NAFs, specialized CCSD and (T) codes	LNO-CCSD(T)/CBS up to 1000 atoms
memory-, disk-, and network-economic code	a few 10(−100) GB memory and disk use on average
independent energy contribution computations	checkpointing, restartable, parallelization
restricted open-shell reference and intermediates	efficient open-shell LMP2 & LNO-CCSD(T)
up to 4-level embedding (see below)	enables protein, solvent, crystal environments
treatment of quasi-redundant basis sets	enables large, diffuse basis sets needed for CBS

**Figure 1:**
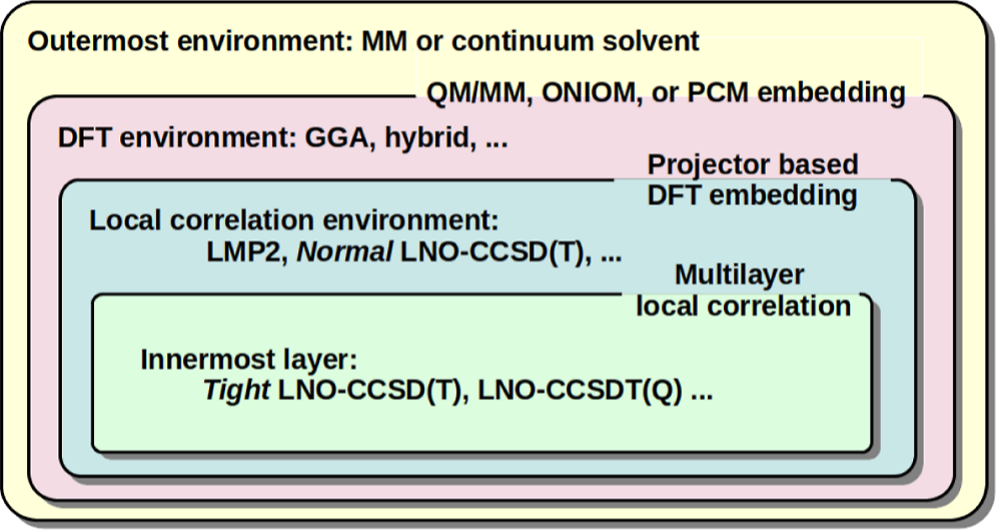
Illustration of the multilayer embedding variations available in the mrcc package.

Calculations can be further accelerated by multilevel approximations and DFT-embedding techniques. Using the mechanical embedding (ME) and electronic embedding (EE) versions of the well-established ONIOM (our own *n*-layered integrated MO and molecular mechanics)^98^ framework or different projector-based embedding (PbE) schemes,^34,35,99^ arbitrary WFT/DFT-in-WFT/DFT methods can be defined. During the development process, we have prioritized minimizing the effort required from the user to set up the calculations. Consequently, a special feature of the ONIOM implementation is that the program handles an arbitrary number of layers in a black-box manner as bonds across subsystem borders and the necessary capping atoms are automatically identified based on the LMOs from the low-level calculation. Additionally, the ONIOM infrastructure can also utilize semiempirical quantum mechanical (SQM) approaches via the mopac(100) and xtb^101,102^ interfaces to push the computationally tractable system sizes further. In contrast to the ONIOM scheme, the PbE approaches are inherently free from capping atoms and utilize a frozen embedding potential for determining the density in the region of interest. Solvation effects can also be accounted for efficiently by continuum models using the pcmsolver(103) library. Finally, for very large systems, further savings can be achieved by the standard quantum mechanics/molecular mechanics (QM/MM) technique via an interface with the amber molecular dynamics program,^104,105^ resulting in the corresponding WFT/DFT-in-WFT/DFT/SQM-in-MM approaches. Among these methods, we point out the possibility of a four-layer method, that is, LNO-CCSD(T)-in-LMP2-in-DFT-D3-in-MM, which can handle systems with tens of thousands of atoms within a reasonable computational time frame.^35^ The multilevel approaches available in the mrcc package are visualized in Figure 1.

To accelerate first- and second-order excited-state calculations, the nearly error-free NAF approximation can be applied,^22,29,36^ while an effective frozen virtual NO approach relying on a state-averaged one-particle density matrix is recommended for iterative second-order methods.^22,29^ To reduce the scaling of the CIS and (TDA-)TDDFT approaches, the LDF approximation can be utilized,^28^ while a domain-based framework using LNOs has been elaborated for the LR-CC2 and ADC(2) methods.^30^ The approximations discussed in this section are collected in Table 4.

**Table 4:** Reduced-Cost and Reduced-Scaling Algorithms Implemented in mrcc

cost-reduction technique	methods	references
dual basis set	HF, KS	(35)
local density fitting	HF, KS	(16 and 26)
	CIS, (TDA-)TDDFT	(28)
natural auxiliary functions	MP2, dRPA, SOSEX, CCSD(T)	(17)
	CIS, (TDA-)TDDFT, CIS(D), DH-TDDFT	(36)
	LR-CC2, ADC(2), CIS(D_∞_)	(22 and 29)
natural orbitals	CC(*n*), CCSD(F12*)(T+)	(27 and 106)
	LR-CC2, ADC(2), CIS(D_∞_)	(22 and 29)
local correlation	MP2, DH-DFT, dRPA, SOSEX	(18, 26, and 107)
	CCSD(T), CC(*n*), CC(*n*)	(16, 19, 23–25, and 108)
	LR-CC2, ADC(2), CIS(D_∞_)	(30)
multilevel methods	WFT/DFT-in-WFT/DFT/SQM-in-MM methods	(34, 35, and 105)

### Methods for Accurate Calculations Approaching the CBS Limit

One of the factors limiting the accuracy of correlation calculations is the potentially slow convergence with the atomic orbital (AO) basis set. The CBS limit is conventionally approximated by utilizing convergent basis set hierarchies in combination with extrapolation techniques.^109−112^ However, for high precision, large basis sets that include high angular momentum functions are still necessary, which significantly increases the computational time required. In this section, we present the advanced and unique features of mrcc implemented to accelerate basis set convergence.

#### Reduced-Cost CC Approaches

A generally applicable way of accelerating post-MP2 calculations is the transformation and truncation of potentially large basis sets. This approach leverages the fact that conventional Gaussian-type AO basis sets are optimized for atoms but can be compressed with minimal loss of accuracy using molecule-specific bases, most commonly NOs.^93,94^ The NO basis is constructed by diagonalizing a model density matrix, typically obtained from the doubles amplitudes of the MP1 wave function. This provides a compact representation of the wave function parameters for post-MP2 methods in terms of the truncated NO basis.^113−116^ The frozen NO (FNO) approximation is predominantly used in quantum chemistry to compress the virtual orbital space; however, mrcc also enables the compression of correlated occupied MOs. While the truncation of the virtual NO space is generally robust and black-box, compressing the occupied space requires more attention to ensure consistency. For instance, when calculating energy differences, it is important to retain the same number of occupied orbitals on both sides of a chemical equation. The combination of the FNO and NAF^17^ approaches proves especially effective as fewer NAFs are required for accurately fitting the ERI tensor in the FNO basis.^117^ These FNO and NAF techniques are available for both the hand-coded CCSD(T) and the general-order CC approaches using closed- and open-shell references. In addition, these techniques also take advantages of symmetry adaptation, which is particularly useful in conjunction with our open-ended CC code, utilizing Abelian point group symmetry.

The error introduced by FNO truncation can be mitigated through an additive MP2-level correction, which comes at negligible computational cost. This correction is based on the difference of the MP2 energies computed with the entire virtual space and with the FNO basis. Furthermore, this MP2-level correction can be extended by exploiting the fact that the particle–particle ladder (PPL) term of CCSD exhibits basis set convergence behavior similar to the MP2 method.^118^ Relying on this observation, a correction scheme has been implemented in mrcc,^119^ where the PPL term of the CCSD energy is scaled by the ratio of full MP2 to FNO-MP2 energy. This multiplicative correction reduces the error of this term by about a factor of 3 and leads to better error cancellation with the uncorrected terms. We have also proposed a size-consistent correction for the PPL and (T) terms, scaling the corresponding correlation energy contributions of each orbital individually.^119^ Alternatively, the systematic convergence of the correlation energy to the untruncated value can be utilized to further reduce the FNO error. In this regard, we introduced an MP2 energy-based linear extrapolation for the correction of the FNO-CCSD(T) correlation energies.^119^ Our detailed benchmark studies on the accuracy of these FNO truncation corrections highlight the superiority of the linear extrapolation correction for the correlation energies; however, for energy differences, the additive MP2 scheme is still recommended.^117,119^

The combination of the FNO and NAF approximations with our extensively optimized CCSD(T) code is also noteworthy.^20^ For the combined approach, a hybrid open multi-processing-message passing interface (OpenMP-MPI) parallelization has been implemented utilizing all permutational symmetry, minimizing network usage, and achieving strong scalability up to several hundred CPU cores with a notable 50–70% peak performance utilization. In contrast to other implementations that primarily focus on the PPL term of CCSD, we have extensively optimized the additional terms of CCSD and introduced a particularly efficient (T) algorithm,^19^ which are especially effective in the compressed FNO basis. Namely, the use of FNOs and NAFs yields reductions in the operation counts of up to fourth and fifth power scaling terms and reductions in the cubic scaling memory requirements.^117^ The combination of the above developments enabled us to push the limits of FNO-CCSD(T) calculations to systems with up to 50 atoms using quadruple-ζ basis sets and up to 75 atoms with triple-ζ basis sets, with negligible FNO errors in energy differences.^117^

#### F12-Based Approaches

F12-based methods also aim to reduce the error associated with one-electron basis sets. Since the basis-set incompleteness error (BSIE) typically scales with the highest angular momentum in a given basis set,^120^ achieving energies near the CBS limit requires large basis sets, which can be costly. Explicitly correlated methods improve convergence by introducing wave function ansätze that explicitly incorporate interelectronic distances.^121−123^

Among the available variants in the literature, the CCSD(F12*) method^63^ has been implemented in mrcc. This explicitly correlated CC approach offers a good balance between accuracy and computational cost, allowing high-quality results even with relatively small basis sets. Additionally, MP2-F12^47−49^ is included as a prerequisite for CCSD(F12*). Both implementations rely extensively on the DF approximation and robust fitting formulas^48,124,125^ to efficiently handle F12-specific integrals.

**Figure 2:**
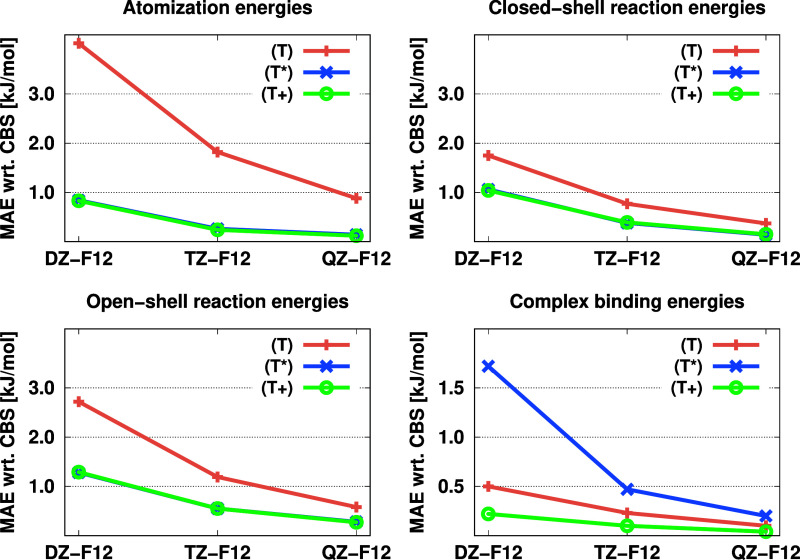
MAEs (in kJ/mol) for the KAW test set and molecular complexes using the (T), (T*), and (T+) contributions with the cc-pV*X*Z-F12 basis sets.

The CCSD(F12*) method can also be combined with perturbative triples corrections. The default method, labeled (T+),^49^ is tailored for explicit correlation and scales the conventional triples contributions for each MO by the ratio of the MP2-F12 to MP2 energy contribution associated with the corresponding orbital. Moreover, the conventional (T) and the (T*) corrections^126^ are also available. However, unlike the (T+) correction, the latter method is not size-consistent. The performance of these triples contributions was benchmarked for the Knizia, Adler, and Werner (KAW) test set^126^ and complexes with aromatic compounds using the cc-pV*X*Z-F12 basis sets (*X*Z-F12 for short).^49^ The mean absolute errors (MAEs) are presented in Figure 2.

As demonstrated, the (T+) approach offers comparable accuracy to the popular (T*) method for the KAW test set. However, based on the results for complexes, the benefits of size consistency are evident as the MAEs obtained with our (T+)method are lower. In light of these results, we recommend using the CCSD(F12*)(T+) method with the cc-pVTZ-F12 basis sets for standard applications.

#### Reduced-Cost F12-Based Approaches

Our explicitly correlated methods can also be combined with the aforementioned cost-reduction techniques, namely the FNO and NAF approximations. Since our explicitly correlated MP2 and CCSD(T) implementations heavily utilize DF, we designed our code to be compatible with NAFs. Explicitly correlated methods require four additional types of integrals beyond the conventional Coulomb-type integrals. However, our reduced-cost approach relies solely on the SVD of the three-center ERIs, which are also essential for fitting the four F12-specific integrals. As a result, this truncation reduces the number of F12-type integrals and enhances both time and memory efficiency by using a single type of fitting basis for all integral types. In CC methods, however, the F12-dependent and the conventional CC intermediates use separate auxiliary bases, requiring separate NAF bases as well. Our third cost-reduction technique, the natural auxiliary basis (NAB) approach, is specific to explicitly correlated approaches.^106,127^ This scheme compresses the complementary auxiliary basis set (CABS) space used in conventional F12-based methods for the resolution of the identity approximations. Similar to the NAF approach, NAB relies on the SVD of three-center ERIs that assemble the F12-type integrals.

**Figure 3:**
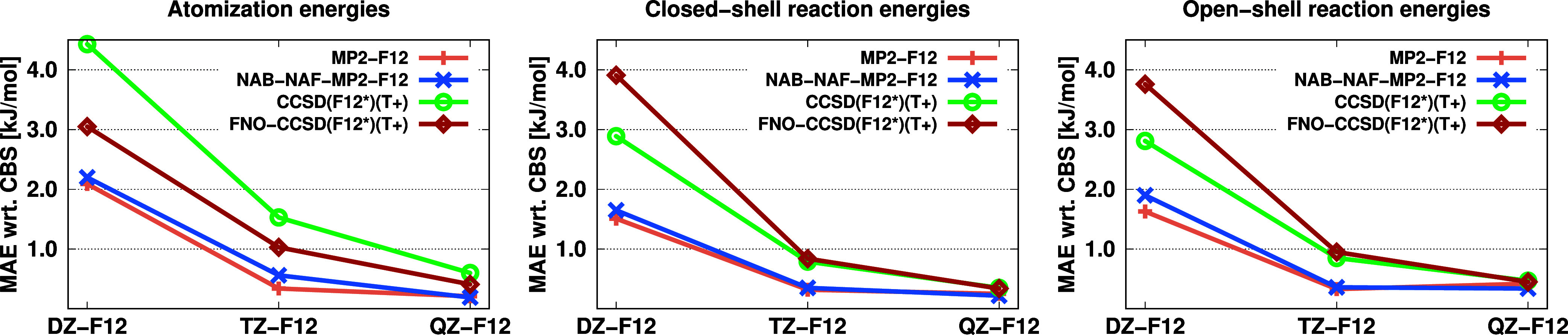
MAEs (in kJ/mol) for the KAW test set using the standard and reduced-cost F12 approaches with the cc-pV*X*Z-F12 basis sets.

These methods can be applied individually or in combination for cost reduction. For MP2-F12, the optimal balance between efficiency and accuracy is achieved with the NAB-NAF scheme.^127^ For explicitly correlated CCSD(T), the FNO-NAB-NAF combination provides significant performance improvements without noticeable accuracy loss if conservative thresholds are chosen.^106^ For simplicity, we will refer to this method as FNO-CCSD(F12*)(T+) from here on, even though all three cost-reduction techniques are being used. For detailed information regarding implementation and algorithmic considerations, we refer readers to the original papers.^106,127^ The performance of the approaches was tested for the KAW benchmark set. The numerical results are presented in Figure 3. As shown, both the NAB-NAF-MP2-F12 and FNO-CCSD(F12*)(T+) methods maintain the accuracy of the exact methods. With the NAB-NAF-MP2-F12 method, we observed speedups of 1.5 to 4 times, while FNO-CCSD(F12*)(T+) offered speedup factors ranging from 3 to 7. These cost-reduction techniques have extended the feasibility of explicitly correlated MP2 and CCSD(T) calculations to systems with up to 61 and 53 atoms, respectively, using reliable basis sets, within relatively short computation times and modest resource requirements.

To further reduce wall-clock times, an OpenMP-MPI parallel implementation has also been developed for our FNO-CCSD(F12*)(T+) scheme.^128^ Specifically, the calculation of three-center integrals, the assembly of intermediates, and the computation of the MP2-F12 energy have been optimized using a two-level scheme, with MPI for the outer loops and OpenMP for the inner loops. At the same time, this significantly improves the memory efficiency of our method as the three-center quantities, which include indices from both the sizable CABS and fitting spaces, can be distributed among the MPI workers, reducing the overall memory load. This parallel implementation is fully compatible with the previously described cost-reduction techniques. Although the truncation of the MO and the various auxiliary function spaces is always performed by the main MPI process, and only the results of the SVD computations are transmitted to the rest of the processes in the MPI communicator, this causes only negligible overhead as the transformation matrices resulting from the cost-reduction approaches are usually of relatively small size. With these improvements, 10-fold speedups have been achieved using 16 MPI processes. Although scaling efficiency decreases with more processes, it remains close to ideal for 2 to 4 MPI processes. These developments have enabled the calculation of a corannulene dimer with the cc-pVTZ-F12 basis set comprising 60 atoms and 2480 AOs, which is the largest system modeled at this level to date. Using 2 MPI processes, the calculation of the F12-specific intermediates required approximately 31 h with 32 CPU cores/MPI process.

#### Basis-Set Corrections

In addition to F12-based methods, an effective procedure for reducing the BSIE of the correlation energy is the density-based basis-set correction (DBBSC) approach, which relies on the RS-DFT formalism.^129,130^ The primary goal of this correction is to account for the missing part of short-range correlation effects that arise due to the incompleteness of the finite one-electron basis set. This is achieved through a local range-separation function that automatically adapts to the spatial nonhomogeneity of the BSIE.

**Figure 4:**
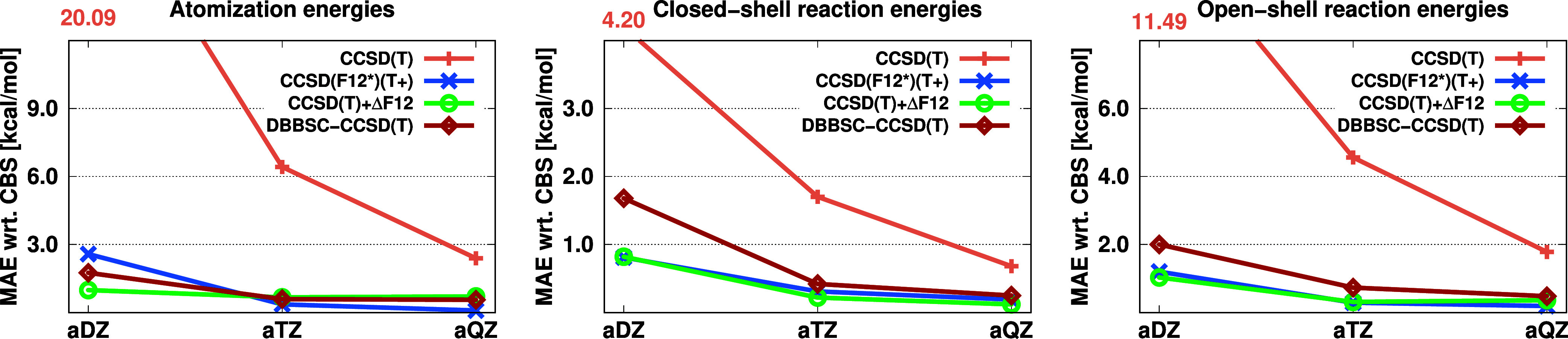
MAEs (in kcal/mol) for the KAW test set using the standard, F12, ΔF12, and DBBSC-CCSD(T) methods with the aug-cc-pV*X*Z basis sets.

In our previous work, an efficient implementation for the DBBSC has been presented^131^ utilizing the DF approximation. This resulted in a cost-effective procedure that scales as the fourth power of the system size. As demonstrated,^129−131^ this approach can efficiently approximate the correlation energy in the CBS limit. However, especially for smaller one-electron basis sets, the HF energy also has a significant BSIE. To address this, the CABS correction^132,133^ was applied. The proposed DBBSC-CCSD(T) method was thoroughly tested in comparison with the F12-based CCSD(F12*)(T+) method. Additionally, a simple incremental approach, denoted as CCSD(T) + ΔF12, was introduced.^131^ In this case, the CCSD(T) total energies are corrected with the CABS correction and explicitly correlated MP2 contributions. The performance of these approaches for the KAW benchmark set with the aug-cc-pV*X*Z basis sets (a*X*Z for short) is depicted in Figure 4.

Inspecting the results, we can conclude that the DBBSC-CCSD(T) approach does not strictly outperform the explicitly correlated CCSD(F12*)(T+) method, although the results are close, particularly when considering those obtained with the aug-cc-pVTZ or larger basis sets. Nevertheless, what makes the DBBSC-CCSD(T) method desirable is that the required wall-clock times are 40% lower compared to CCSD(F12*)(T+).^131^ The incremental CCSD(T) + ΔF12 approach yields surprisingly good results, below 1 kcal/mol even with a double-ζ basis set, while its computational expense is practically identical to that of DBBSC-CCSD(T).

The benefits of the CABS and DBBSC corrections to higher-order CC methods were also examined. Additionally, an effort was made to improve the form of the finite-basis representation of the electron–electron interaction operator, which is conventionally calculated using the HF opposite-spin pair density. To achieve this, higher-order CC densities with systematically increasing excitation levels were employed to define this effective operator.^134^ The performance of the approaches was tested for atomization energies of the Karton benchmark set.^134,135^ The results with the cc-pV*X*Z basis sets (*X*Z for short) are summarized in Table 5. Here, Γ_*M*_ indicates that the two-particle density evaluated with method *M* was employed in the calculation of DBBSC.

**Table 5:** MAEs (in kcal/mol) for the Karton Test Set Using the Standard and Basis-Set Corrected CCSDT and CCSDTQ Methods with Various Opposite-Spin Pair Densities and the cc-pV*X*Z Basis Sets

	CCSDT				CCSDTQ				
basis set	standard	Γ_HF_	Γ_CCSD_	Γ_CCSDT_	standard	Γ_HF_	Γ_CCSD_	Γ_CCSDT_	Γ_CCSDTQ_
DZ	16.31	2.62	3.03	3.03	16.54	2.63	3.04	3.04	3.04
TZ	5.75	0.69	0.87	0.88	5.89	0.75	0.95	0.97	0.98
QZ	2.18	0.30	0.23	0.23	2.28	0.31	0.29	0.29	0.29

As shown, applying basis-set corrections consistently achieves MAEs below 1 kcal/mol for both CC methods, even with a triple-ζ quality basis set. Regarding the use of HF and higher-order CC densities, it can be concluded that applying the CCSD density in high-accuracy calculations, such as CCSDT/QZ, can offer benefits. However, the use of even higher-order CC densities is not justified.

**Figure 5:**
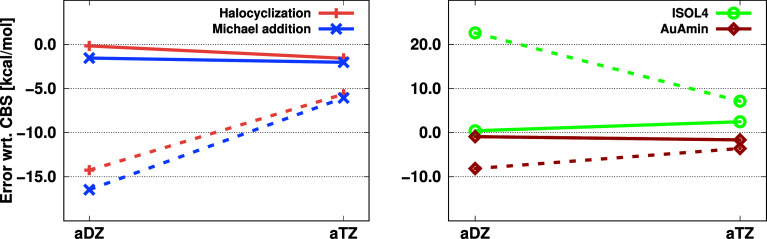
Error (in kcal/mol) of the barrier heights (left) for halocyclization and Michael addition reactions and reaction energies (right) for isomerization and organometallic reactions using the aug-cc-pV*X*Z basis sets. Dashed lines indicate the LNO-CCSD(T) results, while solid lines represent the DBBSC-LNO-CCSD(T) results.

Aiming at large-scale applications, the DBBSC procedure was also implemented in our LNO-CCSD(T) scheme.^25,136^ In this case, the contributions of the range-separation function are decomposed into the sum of contributions from individual LMOs. For these orbitals, a constrained local domain is formed, including all significant interactions, and the corresponding contributions are evaluated only within this compact subspace. To enhance efficiency, prescreening techniques were introduced for grid compression. Furthermore, the LDF approximation^89^ was applied to the calculation of the CABS correction. The efficiency of the proposed DBBSC-LNO-CCSD(T) method was demonstrated through representative examples,^136^ four of which are presented here. That is, the barrier heights for a halocyclization^137^ and an organocatalytic Michael addition reaction,^138^ the isomerization energy for two intermediate steps in a biosynthesis (ISOL4),^139^ and the reaction energy for an organometallic reaction (AuAmin)^140^ were calculated. The errors with respect to high-quality CBS-extrapolated references are presented in Figure 5.

Upon inspecting the results, we concluded that the corrections drastically reduce the BSIE, especially when double-ζ basis sets are used. A minor drawback is that the error of the DBBSC-LNO-CCSD(T) method does not decrease monotonically with increasing basis set size; nonetheless, significant improvements can be achieved with triple-ζ basis sets.

The computational overhead of the DBBSC and CABS corrections was also measured on a single processor with 8 cores. The times required for the time-consuming steps are summarized in Table 6.

**Table 6:** Wall-Clock Times (in min, Upper Panel) Required for the Corresponding Post-HF Steps Using Various Basis Sets^a^

	halocyclization		Michael addition		ISOL4		AuAmin	
step	a(D + d)Z	a(T + d)Z	aDZ	aTZ	aDZ	aTZ	a(D + d)Z	a(T + d)Z
LNO-CCSD(T)	78.2	221.8	323.1	968.8	24.5	76.7	372.0	1162.5
CABS	5.6	16.5	13.7	40.7	5.8	18.0	28.5	70.5
DBBSC	7.2	15.5	25.3	66.8	2.7	5.9	43.2	117.0
overhead	16.4%	14.4%	12.1%	11.1%	34.7%	31.1%	19.3%	16.1%

a For each example, the largest species is shown. a(*X* + d)Z is a shorthand notation for the aug-cc-pV(*X* + d)Z basis sets.

As shown, the calculation of the DBBSC and CABS corrections does not pose significant obstacles. The evaluation of the post-HF steps takes only 10–30% more time compared to LNO-CCSD(T). Accordingly, molecular systems containing several hundreds of atoms can be routinely studied with the DBBSC-LNO-CCSD(T) method, especially since double-ζ basis sets are sufficient for accurate results.

#### Nonatom-Centered or Floating Orbital Methods

To accelerate basis set convergence, particularly for inter- and intramolecular interactions, a wide range of floating orbital (FO) methods are available. We note that these approaches can be used independently or in combination with the reduced-cost standard and F12-based methods, as well as with the DBBSC correction discussed above. While conventional atom-centered AO basis functions efficiently saturate the regions around the nuclei, FO approaches supplement them with additional orbitals located between or around noncovalently interacting subsystems (e.g., monomers), rather than solely on atomic positions.

The currently implemented FO methods in mrcc are summarized in Table 7, which includes details on how these approaches determine the number and position of FOs used.^141^

**Table 7:** FO Methods Implemented in mrcc^b^

	weighted geometric center (WGC)^142,143^	monomer surface grid (MSG)^144^	double layer (DL)^141^	floating orbital grid (FOG)
FO basis/center^a^	3s3p2d1f1g	1s	1s1p(1d)	1s(1p)
number of FO centers	1	∼3–7× system size	∼interacting surface size	∼interacting surface size
number of FOs/center	38	1	4 (9)	1 (4)
position of FOs	WGC of dimer	grid around monomers	interacting region	interacting region
exponents	from ref (142)	optimized in ref (144)	adapted from ref (142)	generated from structure
applicability	dimers of small monomers	only H, C, N, O atoms	no atom type or size restriction	no atom type or size restriction

a Basis set notation *n*_1_s *n*_2_p *n*_3_d... refers to the use of *n*_1_ sets of s, *n*_2_ sets of p, *n*_3_ sets of d, ... functions on each FO center. Parenthesized functions are only employed for non-hydrogen atoms.

b See text for explanation.

The most commonly used FO method in the literature to date (referred to as WGC in Table 7) places only a single FO center at the weighted average of the monomers’ centers of mass or, more recently, at the centers of their intermolecular atom–atom pairs.^142,145^ The simplicity and relatively good performance of this approach have made it the most popular so far; however, its effectiveness decreases for larger complexes containing approximately 10–20 or more atoms.^141^

To increase the number of FO centers, Neogrády and co-workers proposed using a grid of FO centers around the surface of the monomers (referred to as MSG in Table 7).^144^ This scheme is better suited for larger complexes or systems with more than two monomers, although it uses approximately 3–7 times more FO centers than there are atoms in the complex. In addition, currently, its parameters are optimized only for the H, C, N, and O atoms.^144^

**Figure 6:**
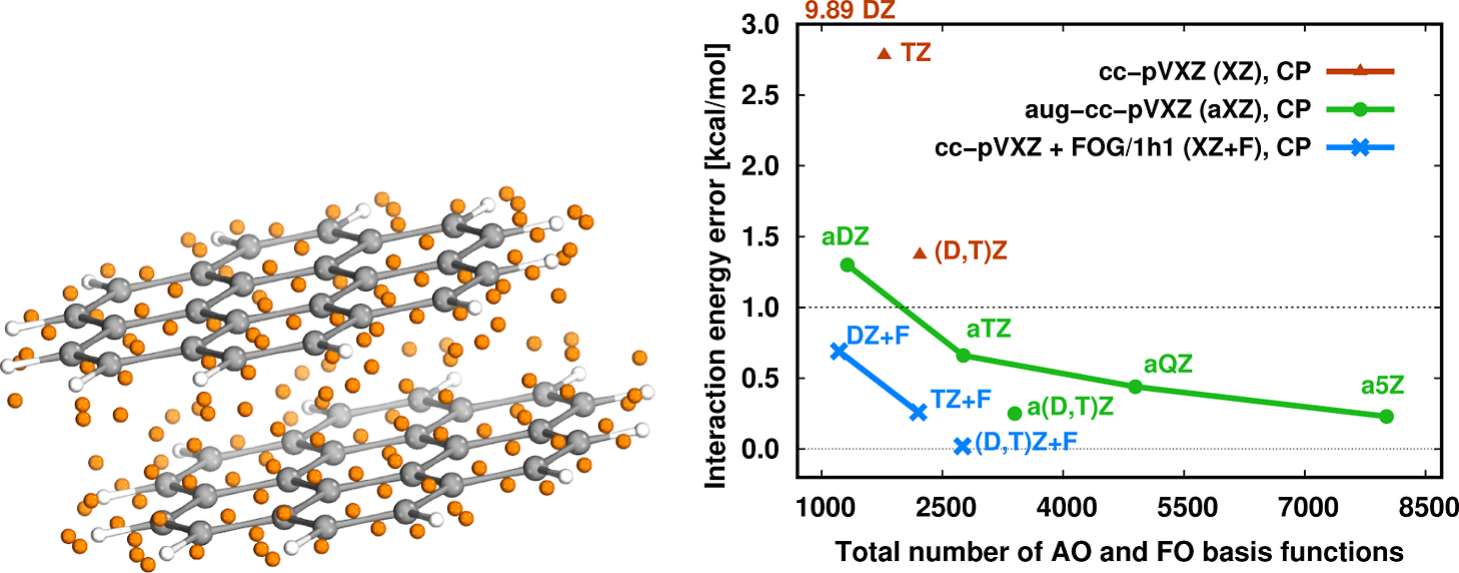
Left: the 154 orange spheres represent the FOG centers for the parallelly displaced coronene dimer. Right: interaction energy errors for counterpoise-corrected LNO-CCSD(T) with the (aug-)cc-pV*X*Z (X = D, T, Q, and 5) basis set with and without FOG basis functions. Reference: counterpoise-corrected LNO-CCSD(T)/aug-cc-pV(Q,5)Z.^146^

To overcome the limitations of monomer size and the lack of parametrization in these FO methods, we proposed combining and enhancing their beneficial properties.^141^ Specifically, we place a grid of FO centers only in the space between the interacting monomers, resulting in a double-layer FO center configuration (referred to as DL in Table 7).^141^ Furthermore, in the floating orbital grid approach (referred to as FOG in Table 7), we automatically generate FO center positions and basis set parameters from the monomer structure and atomic properties. This scheme generates 1 to 2 FO centers for each atom on the monomer surfaces that are relevant for the interaction. An illustrative application is presented for the coronene dimer in Figure 6, where the extrapolated (a)(*X* – 1, *X*)Z results were obtained using the corresponding (a)(*X* – 1)Z and (a)*X*Z basis sets.

A broader statistical analysis on medium-sized dimers shows that the cc-pV*X*Z + FOG and cc-pV*X*Z + DL interaction energies are of comparable quality to cc-pV(*X* + 1)Z or aug-cc-pV*X*Z results.^141^ For larger complexes, such as the coronene dimer, the cc-pV*X*Z + FOG basis set errors are comparable even to aug-cc-pV(*X* + 1)Z for *X* = D and T, while using roughly half as many basis functions (see Figure 6, right). Overall, we can conclude that our FOG scheme is generally more applicable than previous FO methods and performs well for both a larger number of monomers and larger-sized monomers, without restriction on the constituting elements. For more real-life examples, such as supramolecular, catalyst-substrates, and protein–ligand complexes, we refer the reader to ref (141).

### Linear-Scaling Local Correlation Methods for Open-Shell Systems

The use of reliable quantum chemical methods, such as CCSD(T), is limited for large systems due to their significant computational cost. To overcome this, we have developed an asymptotically linear-scaling framework for local correlation methods. The main aspects of these methods are introduced above, while for further details on the closed-shell implementations, we refer the reader to our previous mrcc feature review^37^ and our recent review on local correlation and LNO-based methods.^97^ Here, we focus on the latest extension of these methods to restricted open-shell references,^107,108^ enabling the accurate and efficient modeling of open-shell systems, which are common in a wide range of chemical processes, such as redox reactions, bond breaking, ionization, and electrochemistry.

During the design and implementation of the open-shell variants, our objective was to retain the accuracy, efficiency, and user-friendliness of their closed-shell counterparts (see Table 3). Like the closed-shell variants, our open-shell LMP2 and LNO-CCSD(T) methods are integral-direct, restartable from any stage of the calculation, capable of handling large, near-degenerate AO sets, and support non-Abelian point group symmetry. They can also be embedded in lower-level wave function-based, DFT, or MM environments. Consistency is ensured by recovering the closed-shell result when the open-shell algorithms are applied to closed-shell singlet systems. In addition to ROHF and ROKS, quasi-restricted orbital references are also implemented, which are constructed from unrestricted HF or KS calculations.^107^

To minimize the computational overhead of the open-shell calculations, spin-restricted localized orbitals are employed, while domain-specific restricted, intermediate basis sets are used to ensure that the cost of the most time-consuming integral transformation steps matches that of the closed-shell methods. We introduced a so-called “long-range spin-polarization” approximation, which enables closed-shell algorithms to be applied in domains that interact weakly with the singly occupied orbitals. While this approximation is usually not activated for species of under approximately 50 atoms, for systems with hundreds of atoms, as much as 90% of the domain contributions can be computed using the more efficient closed-shell codes, provided that the singly occupied orbitals are relatively localized to a restricted region of the molecule.^108^

The range of available local correlation methods begins with MP2, a key building block for more advanced local correlation approaches, spin-component-scaled MP2 methods, and DH-DFT functionals.^107^ The use of LMP2 in DH functionals is particularly advantageous because the uncertainty introduced by local approximations is scaled down by the mixing factor of the MP2-like correlation energy term in the exchange–correlation (XC) functional. Based on our experiences, this domain-based approach significantly reduces the computational cost of MP2, making its expenses comparable to or even lower than the those of HF/KS for molecules with a few hundred atoms. More advanced local correlation methods are also available, such as the CC(*n*) and CC(*n* – 1)(*n*) approaches, as well as a highly optimized, high-spin open-shell CCSD(T) implementation.^108^ Several carefully optimized local approximations, including LNOs, NAFs, and a redundancy- and iteration-free perturbative triples correction, extend the applicability of this method to systems with hundreds of atoms and tens of thousands of basis functions.

The accuracy of open-shell LNO-CCSD(T) has been tested against canonical CCSD(T) references across a range of small- to medium-sized radicals, ions, and triplet carbenes. Comparisons between LNO-based and canonical CCSD(T) with the same basis sets showed reassuringly low mean absolute correlation energy errors of 0.05% using the default settings. Such relative correlation energy errors typically correspond to absolute deviations of a few tenths of a kcal/mol for the energy differences,^108^ although larger and more complex molecules often exhibit slower convergence to the canonical limit.^97^

Additionally, a practical feature of our framework is that the local approximations can be systematically converged through a composite threshold hierarchy, that is, Normal, Tight, very Tight... parameter sets. Relying on this, a simple extrapolation scheme was developed^25,97^ to enable faster convergence to the local approximation free limit, referred to as LAF extrapolation. Additionally, a local error estimate and cost-effective composite energy schemes are available to approximate the corresponding canonical CBS limit.^97^ To demonstrate the efficiency of the open-shell methods, key computational details of representative MP2 and CCSD(T) calculations for systems containing 80–565 atoms are provided in Table 8.

**Table 8:** Computational Details for Open-Shell LMP2 and LNO-CCSD(T) Calculations^b^

molecule	vitamin E radical^a^	Cob^II^alamin radical			bicarbonate
no. of atoms	80	179			565
basis set	aug-cc-pV(T + d)Z	def2-TZVP		def2-QZVP	def2-QZVP
no. of AOs	2553	3369		7867	24,712
time of 1 HF iteration	0.05	0.7		2	7
local approximations	Normal	Normal	Tight	Normal	Normal
LMP2 wall-clock time	0.9	4.4	11	27	81
LNO-CCSD(T) wall-clock time	2.9	82	728	156	197
memory demand [GB]	11	18	37	78	105

a Computed using 40 processor cores.

b Wall-clock times (in hours) are measured on a single processor with 20 cores, unless otherwise noted.

**Figure 7:**
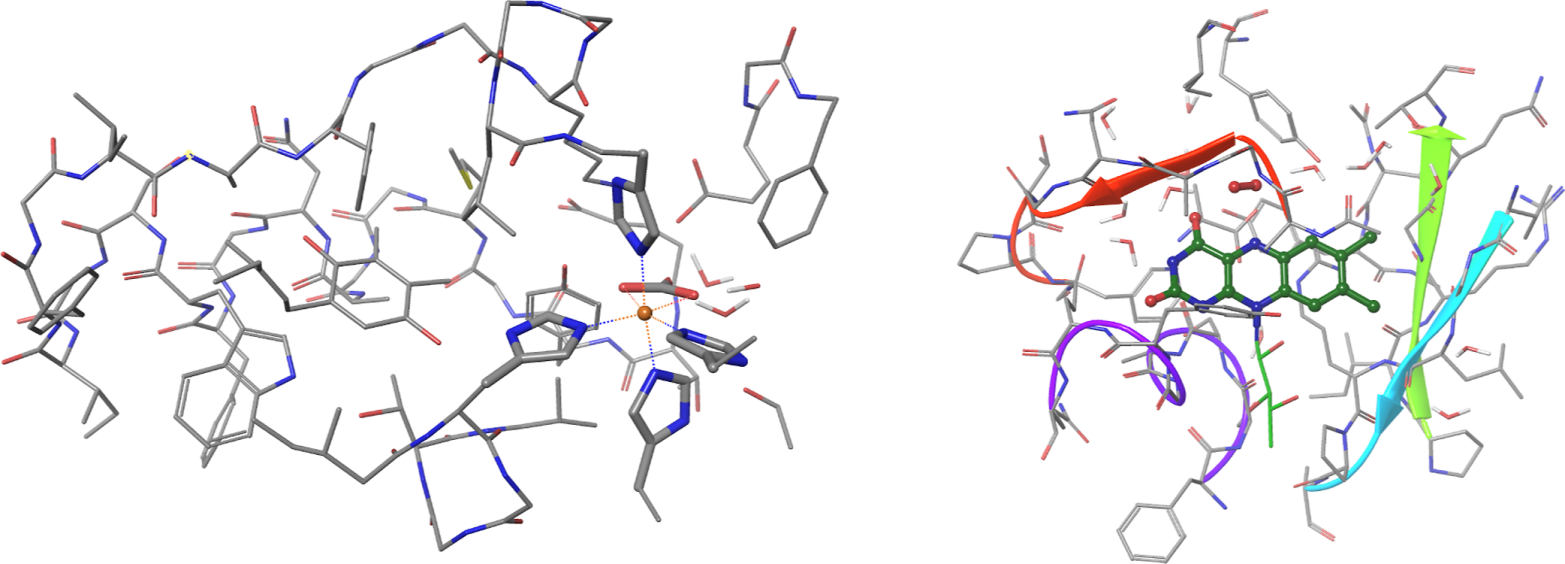
Structures of the two largest protein systems for which local open-shell CCSD(T) energies have been computed. Left: 565-atom model of photosystem II bicarbonate with a sphere denoting the iron(II) center. Right: 601-atom d-amino-acid-oxidase model with the reacting oxygen molecule and central flavin moiety highlighted using balls and sticks.^108^

The results show that the attractive memory requirements of 10–100 GB for LNO-CCSD(T) with Normal settings enable calculations for systems with hundreds of atoms using quadruple-ζ basis sets on a single workstation in a matter of hours to days. For the Cob^II^alamin radical involved in a homolytic bond-breaking reaction of coenzyme B_12_, we present all three calculations required to obtain a high-quality CBS result. To that end, we recommend a composite scheme^97^ that employs Normal and Tight LNO-CCSD(T) calculations with a triple-ζ basis set for the LAF extrapolation toward the canonical CCSD(T)/triple-ζ limit, along with an additional quadruple-ζ calculation used for a CBS-extrapolated basis set correction. Importantly, these three calculations together take roughly 10 times the cost of a single Normal LNO-CCSD(T)/triple-ζ calculation, which is representative of our general experience.^97^ Wall-clock times for a 565-atom model of photosystem II bicarbonate (see Figure 7, left) using a quadruple-ζ basis with nearly 25,000 basis functions are also presented, demonstrating the feasibility of these simulations. In addition to these molecules, open-shell LNO-CCSD(T)/def2-QZVP calculations were also feasible for the spin-state energy gaps of a 175-atom iron(II) complex and the reaction energy for a 601-atom model of d-amino-acid-oxidase (see Figure 7, right). This example represents the largest open-shell LNO-CCSD(T) calculation performed to date, surpassing the previous record by 4.5 times in terms of the number of orbitals.

### Speeding up SCF Calculations

SCF methods play a crucial role in quantum chemistry. On one hand, HF is often used prior to correlation calculations to generate MOs, which, as demonstrated above, often has computational demands comparable to electron correlation methods utilizing local approximations. On the other hand, DFT is one of the most widely used methods in computational chemistry today. As a result, it is important to elaborate techniques that reduce the computational time required for SCF calculations. This section provides details on our developments in this field.

#### Initial Guess

The effect of the initial guess on SCF calculations is a less studied area, despite it being well-known that the initial conditions have a large impact on the convergence of the SCF procedure. A commonly used guess is the superposition of atomic densities (SAD),^147^ where the density matrices from atomic unrestricted HF calculations are used to construct a block-diagonal initial density matrix. A more advanced scheme is to apply the SAD initial guess to an SCF calculation with a “smaller” basis set featuring fewer AOs rather than directly to the target basis set. Then, the density matrix obtained from this small-basis SCF calculation is projected onto the target basis set, followed by a purification step to ensure *n*-representability, and the resulting density matrix is employed as initial guess for the target-basis SCF.^148^ The smaller basis set is often of minimal or double-ζ quality. In addition to conventional schemes, our group has developed a more economical all-electron approach called projected SQM (pSQM) guess.^148^ This method combines SAD for the core orbitals with a density matrix projection technique for the valence orbitals, where the small basis set density matrix is generated using an SQM method. The pSQM approach assumes that the polarized initial density will lead to faster SCF convergence or a higher probability of convergence, while reducing the costs compared to the small basis set calculations for the entire molecule.

To investigate the effects of different initial guess techniques on SCF success rates and iteration steps, extensive benchmarks were completed on various test systems,^148^ including those representing common biochemical bond types. HF and DFT calculations were carried out with various target basis sets, where the initial guesses were taken from SAD, pSQM, and small basis set calculations. The pSQM method utilized the density functional tight binding method GFN2-xTB^101,102,149^ (pGFN), while the STO-3G (pMIN) and def2-SVP (pSVP) basis sets were used for the small basis set calculations. The results showed that pSQM generally reduced the number of SCF iteration steps in comparison with SAD, performing similarly to the more costly pMIN, but pSVP was more advantageous for triple-ζ basis sets. It was also concluded that no significant convergence failures were observed with these initial guess techniques, except for systems containing transition metals, where pSQM showed fewer convergence failures in DFT calculations than SAD or minimal basis set projections.

#### Approximations to SCF Calculations

The DF approximation is one of the most widespread methods to accelerate SCF calculations, although it does not reduce the fourth-power scaling of HF and hybrid DFT calculations due to the formation of the exact exchange matrix. One way to address this is by using the LDF approximation, where domains are constructed for each LMO that contain the relevant AOs and fitting functions. Recently, we have combined the multipole approximation^40,150−153^ with both conventional and local DF-SCF methods.^91^ The most time-consuming step in forming the exchange matrix is the half-transformation of three-center Coulomb integrals. By using multipole approximations, the integrals involving distant orbitals are decomposed so that only the multipole moments of the AO product densities need to be transformed with the MO coefficients.

To test the performance of these schemes, benchmark calculations on various real-life molecules and basis sets were carried out.^91^ The results showed that a speedup of 1.3–1.5 can be achieved for conventional DF-SCF calculations. However, with basis sets containing diffuse functions, this speedup decreased to 1.1–1.2 due to the large spatial extent of these functions. Despite the moderate gains, one of the advantages of the method is that it provides remarkable accuracy, with errors well below 1 μE_h_/atom across all test cases. Consequently, the multipole approximation is recommended when higher accuracy is needed than the LDF approach can provide, but conventional DF-SCF would be too time-consuming. To push the limits further, the approach was combined with the LDF scheme. In this case, the improvements may be even smaller, with speedups of around 1.05–1.2 measured at most, but the accuracy still remained excellent.

Beyond the multipole approximation, we proposed further methods^154^ inspired by the dual basis set approach,^155−160^ where the SCF equations are solved using a less accurate but more economical basis set, followed by a single iteration with a more accurate basis set and a first-order correction to the energy. This approach was extended to use two different fitting basis sets instead of two AO sets. The smaller fitting basis set is employed until SCF convergence, and the final iteration is performed with the larger fitting basis set, with a first-order correction applied to the energy. This scheme is referred to as the dual auxiliary basis approach. Additionally, it is well-known that DF-SCF gives the most accurate results when the Coulomb metric is employed.^161^ In contrast, the overlap metric produces a sparser three-center integral tensor,^162^ making the half-transformation more economical, though the results are less accurate. To combine the advantages of both approaches, a dual metric algorithm was also implemented. In the first step, the overlap metric is used to solve the SCF equations. Then, a single iteration is performed using the Coulomb metric to obtain a more accurate result, which is further enhanced with the first-order correction. In addition to DF, the seminumerical chain of spheres exchange (COSX) approach^163−165^ is another widely used technique to increase the efficiency of SCF methods. While it is standard practice to apply both small and large grids in COSX calculations,^163^ the first-order correction is generally not applied. To assess its impact, we evaluated how much this correction could improve the accuracy of the COSX method.

The performance of these methods was extensively benchmarked using the Adler–Werner test set.^154,166^ For HF total and reaction energies, the dual auxiliary basis method provided accuracy comparable to conventional DF. The dual metric approach, however, showed larger errors, with significant dependence on the basis set; that is, errors were smaller for diffuse basis sets. The seminumerical COSX method exhibited even larger errors in these benchmark calculations. However, for hybrid functionals, the errors were more consistent across the different approaches and basis sets, and significantly smaller compared to HF due to the smaller fraction of the exact exchange contribution in the XC functional. Inspecting the computational requirements, significant improvements can be achieved with these methods. For the dual auxiliary basis approach, speedups of 30–60% were observed, and these gains increased with larger basis set sizes. When the approach is combined with LDF, the speedups were slightly reduced, especially for smaller basis sets, ranging from 10 to 60%. The dual metric approach offered speedups of 50–75%, with the lower values corresponding to calculations using diffuse basis sets.

### Reduced-Cost and Reduced-Scaling Analytic Gradient Calculations

The accurate calculation of gradients with respect to nuclear coordinates is a fundamental yet computationally demanding task in quantum chemistry. Applications that require gradients include, for instance, equilibrium and transition state geometry optimizations as well as molecular dynamics simulations. The following section presents our most recent results regarding gradient development.

#### Analytic Gradients for the LDF-SCF Method

To extend the applicability of the LDF approximation, analytic gradients for the LDF-SCF methods (both HF and KS) have been implemented.^167^ The difficulty of this development arises from the fact that the energy is not variational in this case. Accordingly, a set of coupled-perturbed (CP) HF or CP-KS equations must be solved alongside a set of CP localization equations, in addition to the steps required for a conventional SCF gradient evaluation.

We tested the performance of this approach through benchmark calculations,^167^ which demonstrated that the error resulting from local approximations is negligible. Specifically, the errors in the LDF-SCF gradient are well below 10^–4^ au compared to conventional DF-SCF gradients for both HF and KS-DFT calculations. The optimized equilibrium geometries are also accurate, with typical errors below 10^–3^ Å in bond lengths and around 0.01 to 0.1° for bond and dihedral angles. In spite of the overhead required for solving the CP equations, significant speedups can be achieved by applying the local approximation. The measured wall-clock times are presented in Table 9 for amylose helices containing 4, 8, 16, and 32 glucose units (denoted by amylose4, amylose8, etc.),^168^ the octapeptide angiotensin,^169^ vancomycin,^170^ and a DNA fragment containing two adenine-thymine base pairs (DNA_2_).^171^ The results show that, although calculating LDF-HF gradients is more time-consuming especially for smaller systems, the gains in energy calculations compensate for this, resulting in significantly lower wall-clock times. For instance, a 9-fold speedup was achieved for amylose16 (339 atoms) using the cc-pVTZ basis set, where even the gradient calculations require less time.

**Table 9:** Wall-Clock Times (in min) and Maximum Norms of Gradient Differences (Δ_max_, in au) for Various Test Systems Using Local and Conventional DF-HF with the cc-pVTZ Basis Set

	number of atoms	local DF-HF			conventional DF-HF			Δ_max_
		SCF	gradient	total	SCF	gradient	total	[10^–6^ au]
Amylose4	87	31.7	38.9	70.6	93.3	8.5	101.8	1.1
Amylose8	171	144.7	155.4	300.0	1015.7	100.8	1116.5	3.1
Amylose16	339	577.2	700.2	1277.5	9291.9	1817.4	11109.3	4.2
Amylose32	675	2540.0	7767.7	10307.7				
DNA_2_	128	128.3	144.6	272.9	669.2	49.4	718.6	3.8
angiotensin	146	126.2	151.6	277.8	736.5	61.6	798.2	5.8
vancomycin	176	259.3	314.5	573.8	2082.3	182.3	2264.6	14.3

#### Analytic Gradients for the Huzinaga Quantum Embedding Method

Embedding methods offer an efficient way to handle large systems containing several hundreds or thousands of atoms. In these approaches, the system is divided into smaller subsystems that can be treated at different levels of theory. Thus, for the chemically active subsystem, a more accurate method is used, while for the remaining environment, a faster but probably less accurate lower-level approach is applied. To enable gradient calculations for multilevel methods as well, we have implemented analytic gradients^172^ for the Huzinaga equation-based embedding^34^ approach, which can be considered as the theoretically exact variant of PbE.^173^ Similar to the LDF-SCF methods, the embedding energy is not variational; therefore, the CP-HF/KS and CP localization equations must be solved. Moreover, the resulting equations depend on the specific combination of the applied low- and high-level methods. Developing gradients for all possible combinations of embedding schemes in a black-box manner is not feasible due to the complexity and diversity of these methods. As such, we chose to implement gradients for specific cases, including the DFT-in-DFT, MP2-in-DFT, and DH-DFT-in-DFT embedding schemes.

Our benchmark calculations^172^ demonstrate that small to medium-sized active regions are often sufficient to obtain accurate equilibrium and transition-state structures. Errors are below 10^–1^ Å for bond lengths and 0.5° for bond and dihedral angles, compared to geometries obtained using the high-level method alone. The results also suggest that the high-level method can relax not only the structure of the active subspace but also that of the environment, thereby improving the accuracy of the low-level method for the environment. Inspecting the wall-clock times required, calculations were carried out for a zeolite catalyzed methylation reaction with the Perdew–Burke–Ernzerhof (PBE)^174^ functional and its hybrid (PBE0)^175^ and DH (PBE0-2)^176^ extensions. This system contains 171 atoms, 22 of which were included in the subsystem.^177^ The corresponding wall-clock times are presented in Table 10.

**Table 10:** Timings of Gradient Evaluations for the Zeolite System Using the def2-SVP Basis^a^

low-level method				PBE	PBE
high-level method	PBE	PBE0	PBE0-2	PBE0	PBE0-2
low-level SCF				16.1 (16.7)	21.1 (6.9)
high-level SCF	18.1 (88.4)	733.3 (90.5)	806.0 (8.0)	55.2 (57.5)	81.5 (26.5)
MP2			7331.8 (72.8)		35.6 (11.6)
total energy	18.1 (88.4)	733.3 (90.5)	8137.9 (80.8)	71.3 (74.2)	138.2 (44.9)
CP equations			1412.9 (14.0)	11.8 (12.3)	101.6 (33.0)
gradient	2.4 (11.6)	77.1 (9.5)	520.7 (5.2)	13.0 (13.5)	68.0 (22.1)
total gradient	2.4 (11.6)	77.1 (9.5)	1933.6 (19.2)	24.8 (25.8)	169.6 (55.1)
total wall-clock time	20.5	810.3	10071.5	96.0	307.9

a Wall-clock times (in min) measured on a single processor with 8 cores. Percent contributions of each step to the total wall-clock times are indicated in parentheses.

Our results show that, by using our embedding scheme, a roughly 10-fold speedup can be achieved compared to the high-level methods. In the case of DFT-in-DFT embedding, the energy evaluation consumes the majority of the computation time, whereas the gradient calculation becomes more demanding when DH functionals are employed as the high-level method.

#### Analytic Gradients for DF-MP2 Using NAFs

As demonstrated, the NAF approach efficiently speeds up correlation methods using DF by reducing the size of the auxiliary basis. In our recent work, analytic derivatives for correlation methods that use the NAF approximation were introduced, focusing on MP2.^178^ We presented the detailed implementation of this approach invoking a Lagrangian-based formalism and benchmarked its accuracy and efficiency in geometry optimizations.

To this end, calculations were performed for 30 small molecules from Baker’s test set^178,179^ to determine the optimal truncation threshold for neglecting auxiliary functions after the SVD. Our default cutoff parameter resulted in errors in the calculated gradient elements of below 10^–4^ au, with typical errors in optimized bond lengths (angles) around 10^–4^ Å (10^–4^ degrees). Additionally, the torsional potential energy surface (PES) of C_10_H_22_ was comprehensively investigated, revealing that the number of the retained NAFs can change along the PES for the default truncation value, though only by ±1 across extensive regions of the PES. This causes minor discontinuities in the analytic derivative of the energy, but the PES remains sufficiently smooth to allow for geometry optimizations and the calculation of accurate and quantitatively correct second derivatives. The computational requirements of the NAF-MP2 algorithm was compared to our standard DF-MP2 implementation using a single processor with 6 cores. The wall-clock times show that the integral calculation is overshadowed by other parts of the algorithm for small molecules, leading to only a 5–10% reduction in total wall-clock times. However, for larger systems, the computational cost of the overall algorithm decreases smoothly with system size, achieving a 20% gain for the 92-atom indinavir molecule with the cc-pVTZ basis set. These developments and experiences are important steps toward the analytic gradients of NAF-based CC methods, such as CCSD(T), where significantly greater improvements are anticipated.

#### Analytic DF-CCSD Gradients

In addition to the analytic gradients available for our open-ended CC implementation, recent development has focused on the analytic gradients to our highly optimized DF-CCSD code. In this case, the Lagrange multiplier equations and density matrix computation utilize the DF approximation to avoid I/O operations,^180^ and the t1-transformation technique is applied to both the Lagrange multipliers^181^ and the density matrices^182^ to reduce the number of terms in the corresponding equations. This allows us to extend the optimized algorithmic properties of our DF-CCSD code^20^ to gradient calculations. Through appropriate factorization, approximately the same operation count and asymptotic memory requirements can be achieved for both the CCSD and Lagrange multiplier equations. For example, several of the highly optimized codes for the steepest-scaling terms in the CCSD energy equations, such as the PPL and other sixth-order scaling terms, are reused in the gradient implementation along with its hybrid OpenMP-MPI parallelism. Consequently, evaluating CCSD analytic gradients requires roughly 1 to 2 times the computational cost of CCSD iterations, enabling nuclear gradients and densities to be computed for systems comparable in size to those handled with our DF-CCSD energy implementation (e.g., up to 31 atoms with a quadruple-ζ basis).^20^

### Excited-State Calculations

Building on the available open-ended CC code and the highly optimized implementation of first- and second-order methods, our goal was to enhance both the accuracy and applicability of our excited-state approaches. Due to the inherent complexity of excited-state processes, these developments require careful attention. This section outlines these advancements.

#### Embedding Techniques

To reduce computational costs, multilevel methods such as ONIOM, PbE, and QM/MM approaches are often used for excited-state calculations as well. The mrcc program natively supports ONIOM and PbE methods for excited states, while QM/MM calculations can be performed through the amber-mrcc interface. In the QM/MM and PbE approaches, the excitations are restricted to the active subsystem, whereas the ONIOM algorithm allows for excitations across the entire system. However, system-wide full-spectrum calculations are not recommended, as energy levels of excited states calculated by different methods can easily swap, leading to incorrect assignation and extrapolation.

When excitations are confined to the active subsystem, a cost-effective and relatively accurate option is point-charge embedding (PCE), which, using the ONIOM-EE infrastructure, can also model large systems. In this approach, the environment is represented by point charges during the high-level calculation of the active subsystem. These point charges can either be manually specified or calculated on-the-fly from the electron density of the entire system. Additionally, semiempirical tight-binding DFT methods^101,102,149^ can be used for point-charge determination through the mrcc-xtb interface. Our reduced-scaling excited-state framework^30^ is also noteworthy as it can efficiently model systems with hundreds of atoms. Naturally, this technique addresses some of the limitations of ONIOM methods, such as the use of link atoms for covalently bonded subsystems and the overpolarization of diffuse functions by nearby point charges. Nevertheless, these schemes remain suitable for most biochemical applications, even when the system is divided across hydrogen bonds.

For excited-state calculations involving strongly interacting subsystems, the PbE procedure is recommended. This approach separates the system at the level of MOs, avoiding issues related to link atoms and ensuring that the environment’s potential is not simplified to classical electrostatics. However, the original PbE scheme is not efficient for correlation calculations because it does not separate the virtual subspace between the environment and the active subsystem, which can lead to artificial charge-transfer (CT) states.^183^ Techniques, such as AO basis set truncation^184−186^ and virtual subspace separation,^187,188^ address this issue. In mrcc, the dual-basis approach^35^ is used for basis set truncation, while the subsystem projected AO decomposition (SPADE) algorithm^189^ is employed for virtual subspace separation. For molecular complexes, in many cases, the number of virtual orbitals defined by the SPADE technique matches that from the monomer calculations; however, these virtual orbitals can be significantly distorted, especially if the AO basis set contains diffuse functions.^183^ To mitigate this, Szalay et al. proposed the use of projected AOs for excited-state PbE calculations,^190^ allowing virtual subspace separation while preserving the diffuse components of the virtual MOs. This method has proven essential for homo- and heterodimer PESs, providing excellent results even for Rydberg-type excitations.

The accuracy of these embedding techniques was assessed for ADC(2) using various organic dyes that form hydrogen bonds with solvent molecules.^191,192^ In these benchmark calculations, the dye molecules constituted the active subsystem, while the solvents were represented using various low-level methods, such as PBE, PBE0, and point charges. The MAEs and standard deviations (SDs) are presented in Table 11. Inspecting the results, we conclude that the best performance is attained by the local ADC(2) method.^30^ The MAEs obtained by the PbE-based approaches are also acceptable, though their deviations are somewhat larger, whereas the PCE methods provide the best balance between accuracy and computational cost. It is also noteworthy that all multilevel approaches yield more accurate transition energies than low-level DFT methods. However, the expansion of the active space may be necessary to further improve the accuracy of these methods.

**Table 11:** Errors (in eV) for the Excitation Energies of the XH-27 Test Set^192^ with Respect to ADC(2)/cc-pVDZ References^a^

method	MAE	SD	max.
local ADC(2)	0.023	0.018	0.064
PbE(PBE)	0.074	0.106	0.550
PbE(PBE0)	0.076	0.110	0.584
PCE(PBE)	0.103	0.135	0.412
PCE(PBE0)	0.100	0.129	0.394
ONIOM-ME(PBE)*	0.396	0.494	1.467
ONIOM-ME(PBE0)*	0.122	0.227	0.987
ONIOM-EE(PBE)*	0.325	0.465	1.489
ONIOM-EE(PBE0)*	0.067	0.193	0.991
PBE	1.246	0.582	3.624
PBE0	0.300	0.397	2.236

a The asterisk sign denotes that the statistics for the ONIOM methods are calculated only for the low-energy excitations.

#### RS-DH Functionals

For studying time-dependent properties of extended molecular systems, TDDFT is the most common choice. For semiquantitative accuracy, at least hybrid functionals are recommended; however, the results are often questionable for particular challenging cases,^193,194^ such as Rydberg and CT states, excitations of conjugated systems, or transitions with larger fractions of double excitations. Thus, numerous approaches, including the RS^195,196^ and DH^36,81^ theories, were developed to enable the general usage of TDDFT. In the former scheme, to remedy the wrong long-range behavior of the XC potential, the electron–electron interaction operator is divided into long- and short-range components. For RS hybrid functionals, the long-range (short-range) part of the exchange energy is dominantly covered by the long-range HF (short-range DFT) energy, while the DFT correlation contribution is left unaltered. In the “genuine” DH scheme,^81^ a hybrid TDDFT calculation is performed, and subsequently, the effect of double excitations is added *a posteriori* relying on the CIS(D) method. Recently, an ADC(2)-based ansatz was also developed by our group,^36^ where the effect of the double excitations is treated iteratively.

In our previous work, a simple and robust RS-DH scheme was proposed for excited-state calculations.^21^ This is based on the Coulomb-attenuating method-like decomposition^197^ of the Coulomb potential and the DH ansatz of Kalai and Toulouse,^198^ where both the exchange and correlation contributions are range-separated. This ansatz contains only two adjustable parameters: the range-separation parameter, denoted by μ, and the so-called λ parameter, which can be interpreted as the weight of the wave function methods in the XC energy. One of the advantages of the theory is that well-defined energy expressions are obtained in the limits of the parameters. That is, the standard TDDFT is recovered if μ = 0 and λ = 0. In the μ → ∞ or λ = 1 limits the approach simplifies to the standard CIS(D) method. The one-parameter RS ansatz proposed by Ángyán et al.^199^ is recovered in the 0 < μ < ∞ and λ = 0 case, while a standard DH-like approach is retrieved for μ = 0 and 0 < λ < 1. On top of that, the flexible and efficient implementation facilitates its extension to any combination of exchange and correlation functionals. For that purpose, the local-scaling approximation of Scuseria and co-workers^200^ was adapted.

We note that another attempt has also been made to combine the DH approach with range separation for excited-state calculations.^201^ In this case, a more approximate form of the theory is used, where solely the exchange contributions are range-separated.^202^ This approach recovers the standard DH excitation energies in the μ = 0 limit; however, in the μ → ∞ limit, no other ansatz is recovered. The so-called long-range corrected ansatz was also implemented in the mrcc program package.

The presented approaches can also be combined with spin-scaling techniques.^75,203^ In this case, the opposite-spin and same-spin (D) contributions are scaled separately. Accordingly, this ansatz enables higher flexibility of the energy functional and ensures a more accurate description of the chemical properties. In addition, the computational scaling of the SOS variant can be reduced to *N*^4^. Furthermore, the combination of our spin-scaled RS-DH ansatz and the ADC(2)-based formalism has also been elaborated.^204^ In this case, more accurate excitation energies are expected, especially when the weights of double excitations are relatively large in the excited-state wave function. It is well-known that ADC(2) does not handle double excitations perfectly either; however, its description of them is significantly better than that of CIS(D).^205^ Second, as the perturbative correction is only an energy correction for the CIS(D)-based DH approaches, the transition properties are of just hybrid quality. In contrast, this formalism also allows us to evaluate the transition moments at a higher level, which is essential for the accurate calculation of spectral properties.

The performance of these approaches relying on the TDA approximation has been extensively tested for various benchmark sets including high-quality CC references.^75,204,206^ Here, valence and Rydberg excitation energies (ω) and oscillator strengths (*f*) for the QUEST 1^205,207^ benchmark set are presented, while intra- and intermolecular CT transitions are also assessed for the QUEST CT^208^ and Szalay’s benchmark sets,^209^ respectively. The MAEs and mean relative errors (MREs) are compiled in Table 12.

**Table 12:** MAE (in eV) for the Excitation Energies and MRE for the Oscillator Strengths Using the aug-cc-pVTZ Basis Set^a^

	ω				
method	valence	Rydberg	intra CT	inter CT	*f*
CIS(D)	0.21	0.36	0.35	0.37	0.56
DSD-PBEP86/SCS-CIS(D)	0.11	0.25	0.16	1.08	0.32
PBE0-2/CIS(D)	0.16	0.23	0.22	0.66	0.37
SOS-ωPBEPP86/SOS-CIS(D)	0.11	0.20	0.12	0.66	0.35
RS-PBE-P86/SOS-CIS(D)	0.13	0.23	0.24	0.24	0.43
ADC(2)	0.14	0.31	0.16	0.37	0.19
RS-PBE-P86/SOS-ADC(2)	0.10	0.21	0.12	0.24	0.16

a See text for the benchmark sets employed.

As shown, inspecting the excitation energies, the most balanced performance is attained by RS-PBE-P86/SOS-ADC(2). The obtained results are among the best for valence and Rydberg excitations, while only this method can describe both types of CT excitations with appropriate accuracy. In addition, the relative error of the oscillator strengths is significantly reduced in comparison with the genuine CIS(D)-based DH functionals.

#### Beyond Valence Excitations

Significant advances in modern experimental instruments have turned X-ray spectroscopy into one of the main characterization techniques. The workhorse theoretical approach in this field is the ADC(2) method relying on the core–valence separation (CVS) approximation.^210,211^ In general, to calculate excited states, iterative diagonalization schemes are used, which yield the energetically lowest eigenvalues. As core-excited states are located in the high-energy X-ray region, such calculations for extended systems would be cumbersome. Utilizing the CVS approximation, the couplings between core- and valence-excited states can be neglected *a priori*, allowing the equations to be solved only for the targeted core-excited space. The formalism was also extended to hybrid TDDFT calculations;^212^ however, as expected, the well-known self-interaction problem leads to a strong underestimation of the energies of core-excited states.

In our previous work, the combination of the CVS approximation with the DH theory was elaborated.^213^ In this case, a CVS-CIS(D)-based formalism for genuine DH-TDDFT was presented, while a more advanced ansatz was also elaborated, combining the CVS-ADC(2) method and our ADC(2)-based DH formalism. The performance of the most popular DH functionals was benchmarked against high-quality CC results using the recently proposed XABOOM^214^ test set. The numerical results are collected in Table 13.

**Table 13:** Overall Performance of the Methods for Different K-Edge Excitations^a^

	ω		*f*	
method	MAE	SD	MRE	SD
CIS(D)	1.94	0.54	82	13
PBE0-2/CIS(D)	1.00	0.31	56	10
SOS-PBE0-2/SOS-CIS(D)	0.76	0.20	56	10
DSD-PBEP86/SCS-CIS(D)	3.21	0.35	44	10
B2GPPLYP/CIS(D)	4.02	0.36	40	15
ADC(2)	1.88	0.45	19	15
PBE0-2/ADC(2)	0.69	0.25	11	10
SOS-PBE0-2/SOS-ADC(2)	0.82	0.14	11	5
DSD-PBEP86/SCS-ADC(2)	2.85	0.28	13	11
B2GPPLYP/ADC(2)	3.72	0.31	15	13

a MAE(ω) and SD(ω) are given in eV, while MRE(*f*) and SD(*f*) are given in %.

The results can be discussed from two perspectives. First, the benefits of the CVS-ADC(2)-based formalism can be considered in comparison with the CVS-CIS(D)-based one. Second, the effects of the CVS-DH formalism can be assessed compared with the corresponding wave function-based approaches. Inspecting the excitation energies, these benchmark calculations show that the CVS-CIS(D)-based approaches are highly competitive with the more advanced CVS-ADC(2)-based methods. This finding is valid whether accuracy or precision is considered. On the other hand, as expected, huge differences were observed in the oscillator strengths. In this case, the accuracy of the CVS-ADC(2)-based methods is considerably better; however, the deviations are acceptable for the CVS-CIS(D)-based functionals as well. Concerning the performance of the CVS-DH approaches, we can conclude that the most outstanding results are attained by PBE0-2, while its SOS variant also seems to be reliable. For these approaches, significant improvements are realized compared with the corresponding wave function-based counterparts.

In addition, our reduced-cost scheme based on the frozen virtual NO and NAF approximations was extended to CVS-ADC(2) calculations.^22,215^ The errors introduced by this approach were comprehensively analyzed for more than 200 excitation energies and 80 oscillator strengths. As demonstrated, computational requirements can be significantly reduced, albeit with the introduction of a moderate error. That is, the MAE for the excitation energies, being lower than 0.20 eV, is an order of magnitude smaller than the intrinsic error of CVS-ADC(2), while the MRE for the oscillator strengths is between 0.06 and 0.08, which is still acceptable. At the same time, an overall 7-fold speedup is obtained in the wall-clock times, with dramatic reductions in the memory requirements. We note that this scheme can be freely combined with the CVS-ADC(2)-based DH formalism, where the errors are expected to be even smaller.

Vertical ionization potentials (VIPs) and electron affinities (VEAs) are also crucial parameters characterizing the electronic structure of molecular systems. In our previous work, the TDDFT formalism was extended to directly calculate VIPs and VEAs within the DH theory.^216^ To this end, first, the generalization of CIS(D) to such transitions was elaborated. As demonstrated, the obtained equations retrieve the second-order self-energies in the diagonal and frequency-independent approximations.^217^ Thereafter, the extension to the genuine DH formalism is fairly straightforward. Additionally, relying on the non-Dyson ADC(2) approach, an ADC(2)-based DH analogue was also elaborated to calculate ionized and electron-attached states. To inspect the accuracy of the best DH functionals, up-to-date test sets were used with high-level CC references.^218–220^ The measures for the overall performance of the approaches are collected in Table 14.

**Table 14:** MAE (in eV) for VIPs and VEAs for the Best Performers

method	VIP	VEA
CIS(D)	0.83	0.49
SOS-CIS(D)	0.33	0.15
RS-PBE-P86/SOS-CIS(D)	0.31	0.40
SOS-ωPBEPP86/SOS-CIS(D)	0.23	0.55
SOS-PBE0-2/SOS-CIS(D)	0.35	0.52
ADC(2)	0.59	0.43
SOS-ADC(2)	0.21	0.14
RS-PBE-P86/SOS-ADC(2)	0.18	0.35
SOS-PBE0-2/SOS-ADC(2)	0.30	0.50

Analyzing the results, we can conclude that the most reliable method is the SOS-ADC(2) approach. Among the functionals, the most robust performance is attained by the SOS-ADC(2)-based RS-PBE-P86 method. For ionization potentials, it outperforms SOS-ADC(2). This method also provides the lowest overall MAE for VEAs; however, the accuracy is far below that obtained for SOS-ADC(2). Nonetheless, the advantages of the more advanced ADC(2)-based DH formalism are clearly demonstrated.

## Conclusions and Outlook

Future developments will continue to be primarily driven by our research interest; however, collaborations with users will also be maintained, during which any emerging needs and requests will be considered for implementation in the code. The developing direction will remain guided by our original philosophy: accurate and efficient calculations for large-scale applications.

Efforts will be made to expand the range of physical and chemical quantities that can be computed with the program. Accordingly, highly optimized analytic gradient codes will be written for ground- and excited-state methods. These will be combined with our well-established reduced-cost and reduced-scaling techniques. Our primary goal is to implement an efficient procedure for the calculation of LNO-CCSD(T) gradients, and significant development opportunities are offered by TDDFT and ADC(2) gradient codes. Additionally, we plan to extend our popular methods to open-shell systems.

Furthermore, significant progress is expected in the field of combined WFT-DFT methods. Nonconventional techniques will be developed to further enhance the accuracy of these approaches. The performance of the DBBSC method will be improved, and correlation energy density-based methods will be designed. These features will be available in future versions of mrcc.

The development team is intended to be expanded further. Enthusiastic and dedicated young colleagues are encouraged to join the group. Numerous national and international grants are available to finance these positions. Additionally, collaborations with theory- and experiment-oriented groups are welcome, particularly for exploring complex molecular interactions, reaction mechanisms, and photochemical processes.
